# The influence of conditioned stimuli on [^11^C]-(+)-PHNO PET binding in tobacco smokers after a one week abstinence

**DOI:** 10.1038/s41598-021-90915-y

**Published:** 2021-06-03

**Authors:** Patricia Di Ciano, Harriet de Wit, Esmaeil Mansouri, Sylvain Houle, Isabelle Boileau, Bernard Le Foll

**Affiliations:** 1 Translational Addiction Research Laboratory, Centre for Addiction and Mental Health, Toronto, Canada; 2 Addictions Division, Centre for Addiction and Mental Health, 33 Russell Street, Toronto, ON M5S 2S1 Canada; 3 Addiction Imaging Research Group, Centre for Addiction and Mental Health, Toronto, Canada; 4 Campbell Family Mental Health Research Institute, Centre for Addiction and Mental Health, Toronto, Canada; 5 Brain Health Imaging Centre, Centre for Addiction and Mental Health, 250 College Street, Toronto, ON M5T 1R Canada; 6 Department of Pharmacology and Toxicology, University of Toronto, Toronto, Canada; 7 Department of Psychiatry, University of Toronto, Toronto, Canada; 8 Institute of Medical Sciences, University of Toronto, Toronto, Canada; 9 University of Chicago, Chicago, IL USA; 10 Institute for Mental Health Policy Research, Centre for Addiction and Mental Health, Toronto, Canada

**Keywords:** Motivation, Reward

## Abstract

Stimuli previously paired with drugs of dependence can produce cravings that are associated with increased dopamine (DA) levels in limbic and striatal brain areas. Positron Emission Tomography (PET) imaging with [^11^C]-(+)-PHNO allows for a sensitive measurement of changes in DA levels. The purpose of the present study was to investigate changes in DA levels, measured with PET imaging with [^11^C]-(+)-PHNO, in regions of interest in smokers who had maintained abstinence for 7–10 days. Participants (N = 10) underwent two PET scans on separate days, during which they viewed either smoking-related or neutral images, in counterbalanced order. Craving was measured with the 12-item Tobacco Craving Questionnaire (TCQ) and the Questionnaire on Smoking Urges-Brief (QSU-B). Compared to neutral cues, smoking cues did not increase craving. There were no changes in [^11^C]-(+)-PHNO binding in the cue condition compared to the neutral condition for most regions of interest (ventral pallidum, globus pallidus, limbic striatum, associative striatum, sensorimotor striatum). However, binding potential in the substantia nigra was greater in the smoking-cue condition, indicating decreased synaptic dopamine. There is a potential change of DA level occurring in midbrain following the presentation of smoking-related cues. However, this preliminary finding would need to be validated with a larger sample.

## Introduction

Cues previously paired with drugs of abuse are known to have powerful effects on drug-seeking and relapse in animals^1,2^. In particular, cues are believed to be critical for the maintenance of nicotine-seeking responses^3,4^. In human smokers, drug-related cues produce greater ratings of ‘craving’ as compared to neutral cues^5^. Converging evidence implicates dopamine (DA) and DA receptors as critical in the development of substance use disorders^6^, and in behaviors mediated by drug-paired cues. For example, DA is increased in the nucleus accumbens of rats following the presentation of drug-related non-contingent cues^7–11^.

One powerful non-invasive means of measuring DA in humans is through the use of positron emission tomography (PET) using radioligands selective for DA such as [^11^C]raclopride. Using PET imaging, it has been shown that drug-paired cues can increase DA levels in both dependent and non-dependent stimulant users. In one study, it was shown that cues paired with amphetamine can decrease [^11^C]raclopride binding (increase DA) in the ventral striatum of healthy participants^12^. In dependent and non-dependent cocaine users, [^11^C]raclopride binding was decreased in the dorsal caudate and dorsal putamen during cue presentation^13–15^. Similarly, cocaine-related cues also decreased [^18^F]fallypride in all regions of interest, including the ventral limbic striatum, amygdala, hippocampus, associative striatum and sensorimotor striatum, in dependent patients^16^.

We have conducted a previous study examining responses to smoking cues using PET, but found that smoking cues failed to either increase ‘craving’ or change [^11^C]-(+)-PHNO binding^17^. However, there were several reasons why we may not have detected effects in the previous study. Most importantly, participants were not abstinent from smoking, smoking their previous cigarette on average about 72 min prior to the PET scan. Indeed, in reports of cue-elicited changes in [^11^C]raclopride binding in stimulant users, participants had not used drugs for at least 12 h, and sometimes longer^12,14–16^. Thus, the cues in our non-abstinent smokers may not have been sufficiently salient. Moreover, it is known that the intensity of cue-induced ‘craving’ can increase over long periods abstinence^18^ in smokers^19^. This raised the possibility that smoking cues may affect DA levels only after a period of abstinence period.

DA levels can be studied using PET with either [^11^C]-(+)-PHNO or [^11^C]-raclopride. We used [^11^C]-(+)-PHNO because there is some evidence that it is sensitive to smaller changes in synaptic DA levels. One study in cats^20^ reported greater sensitivity of [^11^C]-(+)-PHNO compared to [^11^C]-raclopride in assessing the dose–effect of amphetamine (0.1, 0.5 and 2 mg/kg; i.v.). Similar findings have been obtained with a *d*-amphetamine challenge in human participants^21^, with better sensitivity of [^11^C]-(+)-PHNO vs [^11^C]-raclopride^22^. In addition, we have recently reported that the impact of smoking on [^11^C]-(+)-PHNO binding is much higher compared to what has been previously reported with [^11^C]-raclopride (increase of 50% of sensitivity)^23,24^.

The purpose of the present study was to investigate [^11^C]-(+)-PHNO binding in regions of interest after presentation of smoking-related cues as compared to neutral cues in abstinent smokers. Regular smokers abstained from smoking for 7–10 days prior to two PET scans. The scans were conducted 1–3 days apart, smoking-related cues and neutral cues tested in counterbalanced order. We hypothesized that subjects would report greater craving after smoking-related cues than neutral cues, and that [^11^C]-(+)-PHNO binding would be lower (corresponding to higher DA levels) in the smoking-cue condition as compared to the neutral-cue condition. The regions of interest that were examined were the substantia nigra (SN), ventral pallidum (VP), globus pallidus (GP), limbic striatum (LST), associative striatum (AST) and sensorimotor striatum (SMST).

## Results

### Demographics

Ten participants completed the study (5 male, 5 female). Their mean age was 44.5 years (range 24–58). Participants had an average Fagerstrom Test for Nicotine Dependence (FTND) score of 5.3 (± 0.47) and smoked on average 16.3 (± 1.31) cigarettes a day for the last year (range 10–20). All participants were cigarette smokers, and no vapers. There were no differences between neutral and smoking cue scans in the carbon monoxide (CO) levels prior to the scan or the time since their last cigarette. Of the 10 participants, 2 had never tried cannabis, 3 had tried it a few times, 2 had not used it in a few years, and 3 had used it in the past 90 days. On the day of the PET scans, all participants screened negative for psychoactive drugs in a urine toxicology test (cocaine, amphetamine, methamphetamine, cannabis, opiates, methadone, bariturates, benzodiazepenes, oxycodone, PCP, buprenorphine, tricyclic antidepressants, MDMA). As well, the mean Mass Injected, Corrected Activity and Specific Activity did not vary significantly between the scans with the smoking-cue or neutral cue presentation. Salivary cortisol was not analysed due to problems of collection of samples in the scanner. For one condition, there was insufficient saliva collected for 30% of the samples. See Table 1.

**Table 1:** Mean ± SEM descriptive characteristics of participants.

Descriptive	Mean ± SEM	p value	
Ratio Male: Female	5:5	n/a	
Age (years)	44.5 ± 3.34 (24 to 58)	n/a	
Fagerstrom test for nicotine dependence (FTND)	5.3 ± 0.47 (3 to 7)	n/a	
Cigarettes per day (CPD)	16.3 ± 1.31 (10 to 20)	n/a	

	Smoking	Neutral	
Expired carbon monoxide (CO)	2.7 ± 0.42	2.6 ± 0.45	p > 0.05
Time since last cigarette (days)	8.6 ± 0.45	8.3 ± 0.45	p > 0.05
Mass radioligand injected (μg)	1.65 ± 0.12	1.65 ± 0.13	p > 0.05
Corrected activity (mCi)	10.47 ± 0.22	10.58 ± 0.21	p > 0.05
Specific activity at time of injection (mCi/μmol)	2528.7 ± 163.23	2549.8 ± 179.22	p > 0.05

P values correspond to the results of paired samples *t*-tests. Range is provided in brackets.

### Binding potential

**Figure 1:**
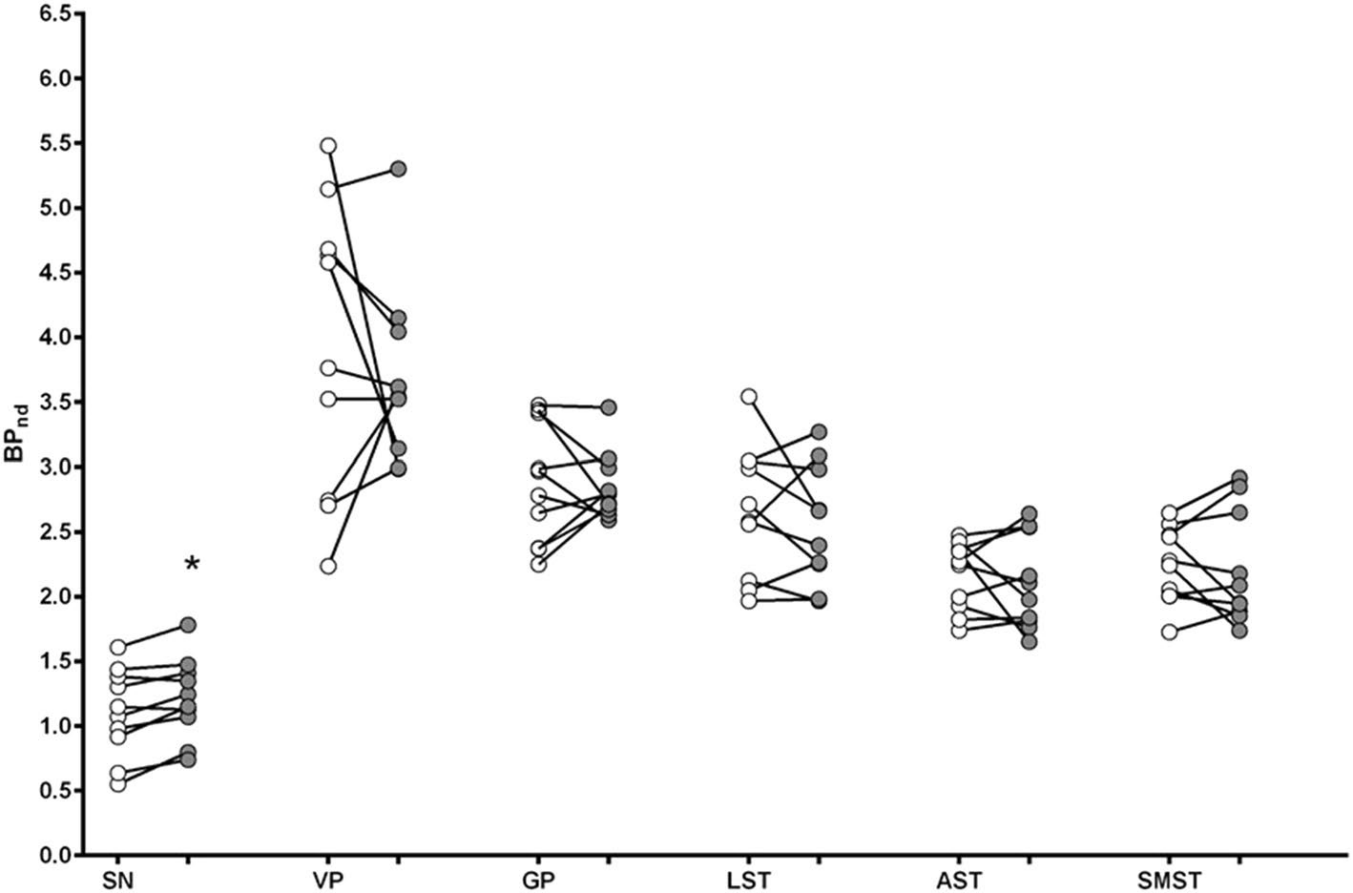
Binding potential (BP_ND_) for the individual participants after presentation of the neutral cue (open symbols) or smoking cue (dark symbols). The horizontal line represents the mean of the group. *SN* substantia nigra; *VP* ventral pallidum; *GP* globus pallidus; *LST* limbic striatum; *AST* associative striatum; *SMST* sensorimotor striatum. *p < 0.0083, neutral cue different from smoking cue.

BP_ND_ during the smoking cue was similar to that measured in the presence of the neutral cue for most ROIs (VP: -0.05%; GP: 0.8%; LST: 2.9%; AST: 2.8%; SMST: − 1.8%). For the SN, BP_ND_ was increased in the presence of the smoking cue (12.9 ± 4.5%). A Cue x ROI ANOVA found a significant interaction (F(5, 45) = 67.339, p < 0.001, *ƞ*_p_^2^ = 0.882). Bonferroni-corrected two-tailed paired samples *t*-tests on the effect of Cue for each ROI found a significant effect for the SN (t(9) = − 3.556, p = 0.006), which survived the correction for multiple comparisons (corrected p value of 0.0083). See Fig. 1. Given the small sample size, it was of interest to determine the power needed to see a 5% change in BP_ND_ between the neutral and cue conditions. The marginal means for the neutral and smoking conditions, respectively, was 2.37 and 2.31, meaning that a 5% difference is an effect size of 0.12. An effect size of 0.12 is equivalent to a Cohen’s d of 0.25, which is a small effect.

### Craving

Craving did not differ in the smoking vs neutral conditions, on any scale (repeated-measures ANOVAs: QSU1: (F(4, 36) = 0.523, p > 0.05, *ƞ*_p_^2^: 0.055; QSU2: F(4, 36) = 0.607, p > 0.05, *ƞ*_p_^2^ = 0.063; TCQ1: (F(4, 36) = 0.658, p > 0.05, *ƞ*_p_^2^: 0.068; TCQ2: (F(4, 36) = 1.44, p > 0.05, *ƞ*_p_^2^: 0.138; TCQ3: (F(4, 36) = 0.425, p > 0.05, *ƞ*_p_^2^: 0.045; TCQ4: (F(4, 36) = 1.368, p > 0.05, *ƞ*_p_^2^: 0.132; PANASpos:F(2,18) = 2.236, p > 0.05, *ƞ*_p_^2^ = 0.199; PANASneg (F(2, 18) = 2.913, p = 0.08, *ƞ*_p_^2=^0.245).

## Discussion

The purpose of the present study was to investigate cue-induced changes in DA in regions of interest in smokers who were abstinent for 7–10 days. Analysis of differences in BP_ND_ between the smoking and neutral cue condition revealed that BP_ND_ was higher in the presence of the smoking cue in the SN, corresponding to decreased DA levels in this condition. The smoking and neutral cues did not result in different levels of craving.

In the present study we did not find any significant increases in craving in response to the smoking-related cues. With the small sample size, it is possible that the present study was not powered to see any cue-induced craving. However, the small effect sizes observed in the present study argue against this explanation, and increasing the sample size may not have yielded different results. As mentioned above, previous studies employed different types of cue presentation and thus it is possible that more salient cues may have been needed, especially considering that participants were also scanned during most of the cue presentations. Nevertheless, the findings with the craving questionnaire are consistent with previous findings^19^ that used similar cue presentations. In this latter study, cue-induced craving was observed at 5 weeks of abstinence and thus it is possible that under the present conditions a longer ‘incubation’ period is required to observe cue-induced changes. It should also be mentioned that previous studies measured craving with visual analog scales while in the present study the validated QSU-B and TCQ were used. It is possible that these assessments are measuring different aspects of craving.

Despite the lack of cue-induced craving in the present study, DA levels in the SN were lower in the smoking-cue condition compared to the neutral-cue condition. This finding is unexpected given the previous findings of increases in DA following the presentation of drug-related cues^12–16^. However, previous studies reported increased DA in terminal regions of the DA system, while we detected effects in the SN, a cell body region, an area not examined in previous studies^12,14–16^. Despite the lack of cue-induced craving measured in the present study, it appears that the experimental situation produced some changes in DA. Future studies will need to assess craving using a variety of different approaches. In sum, this study suggests that there may be dynamic changes in DA in various brain regions in response to cues.

It is important to consider the SN as part of a larger DA system controlling motor output. That is, the SN is known to project to the dorsal striatum, while the terminal regions of the LST have their cell bodies in the ventral tegmental area^25^. It is generally accepted that there are two types of motor responses to stimuli, those that are goal-directed and flexible, and those that are habitual^26^. In this regard, it has been proposed that the SN-dorsal striatum and ventral-tegmental-LST pathways control habitual and goal-directed responding, respectively^27^. Animal studies have shown that DA levels are increased in the nucleus accumbens (LST) following the presentation of passively presented cues, but not after conditioned reinforcers, which are earned through operant responses^7–9,28^. Indeed, operant responding for conditioned stimuli may represent a more habitual form of behavior^29^, which may involve the dorsal striatum-SN circuit^30^. This form of instrumental responding for cues has been shown to be especially important in nicotine reinforcement^3^. Thus, it is possible that the cue presentations in the present study may have activated a habit system and motor responses that were not measured in the present study. Future investigations will need to consider different types of stimulus control and behavioural output.

Indeed, the nature of the craving questionnaires and cues used in the present study may explain why we did not observe any increases in DA in terminal regions, as reported in previous studies^12–16^. Previous studies used different types of cues^13–15^, including lengthy videos or written narratives by the subjects about their drug use^16^. Finally, one study compared a scan in the presence of amphetamine to that after a placebo^12^. In the present study, participants were shown pictures of smoking-related scenes, but no videos or personal narratives. Thus, it may be that cues used in PET studies must be especially salient. Indeed, in our previous study we also did not find any effects of cues on DA when the cues were presented as images^17^. PET scans require bolus intravenous injections, and in the present study participants also had to wear masks. Added to this, participants must remain immobile for up to an hour or more. Thus, it may be that care should be taken in selecting salient cues.

PET imaging with [^11^C]-(+)-PHNO provides a sensitive measure of DA levels in regions of interest and also provides a region-dependent measure of levels of D2 and D3 receptors. Previous studies have shown that binding of [^11^C]-(+)-PHNO in the SN is largely due to binding to D3 receptors, where binding in the VP and GP provide intermediate binding to D3 receptors^31^. By comparison, binding in the AST, SMST, DC and DS are largely due to binding to D2 receptors^31^. Thus, it can be suggested that differences in BP_ND_ between the smoking and neutral cue conditions may reflect differences in the levels of D2 or D3 receptors during these two conditions. This is unlikely because the order of the neutral and smoking cue conditions was counterbalanced, and thus differences do not reflect changes in receptor levels. Thus, differences in [^11^C]-(+)-PHNO binding between the two conditions in this study likely reflects a difference in levels of DA.

### Limitations

This study had limitations. First, the sample size is small, with only 10 participants. It was difficult for participants to maintain abstinence for more than a few days, and a high attrition rate lead to a smaller sample than planned. Future large-scale studies would need to plan for the enrollment necessary to achieve a larger final sample size. However, it should be mentioned that the effect sizes for the craving measures were very small, suggesting that differences in craving between the two cue conditions may not have been found, even with a larger sample size. A second limitation is the fact that only the one week of abstinence was studied. ‘Incubation’ of craving has been found up following up to five weeks of abstinence^19^ and thus it is possible that significant cue-induced craving and differences in DA levels would have been found with a lengthier abstinence. A third limitation is related to the test–retest variability of [11C]-(+)-PHNO. Even though [11C]-(+)-PHNO is speculated to have greater displacement potential, its test–retest variability is 9%, which is higher than that for [11]raclopride (4–6%). Thus, this potentially limits the detection of smaller effects^32^. In this regard, in this study, the power to detect a 5% change in BPND is very low, with a Cohen’s d of 0.25.

## Methods

### Participants

All procedures were approved by the Centre for Addiction and Mental Health Research Ethics Board and complied with the 1975 Helsinki Declaration (5th revision, 2000). Men and women were recruited from the community, provided written informed consent, and participated in a comprehensive screening interview. All met the following criteria: (1) Adults of any ethnic origin 19 years of age or older; (2) smoke 10 or more cigarettes a day; (3) no medical conditions counter-indicating participation; (4) no lifetime diagnosis or treatment for psychosis or mania; (5) no psychiatric diagnoses or treatment in the past year; (6) no current use of psychiatric medications; (7) no current substance dependence except nicotine; (8) not pregnant; (9) must be able to understand the study procedures; (10) no presence of metal objects in the body or implanted electronic devices that preclude safe MRI scanning; and (11) no claustrophobia or other counter-indications with MRI or PET scanning.

### Procedure

Participants were recruited by word-of-mouth, advertisements in local newspapers, social media, through posters and from referral from other studies. After an initial phone screen, eligibility was assessed after obtaining signed informed consent. Eligible participants underwent 2 PET scans after being asked to refrain from smoking for a period of 7–10 days. Before the sessions, participants reported to the lab at least 3 times a week to verify abstinence with expired carbon monoxide (CO) levels below 10 p.p.m and urine cotinine levels below 500 ng/ml. All participants screened negative for drugs of abuse on the day of the scan, and had CO levels below 10 ppm and cotinine levels below 500 ng/ml. During the PET sessions, participants viewed smoking-related or neutral cues, in counterbalanced order. Cues, consisting of a 5-min slide show, were presented four times: 35 min prior to the scan (cue 1), at the start of the scan (cue 2), and 15 (cue 3) and 30 min (cue 4) into the scan. Cue images were drawn from the ISIS smoking image series, and supplemented with additional cues. Each slide was presented for 5 s and alternated between an image and central fixation cross in the slide, for a total of 60 slides per show (30 images and 30 blank with crosses). When presented with the slide show 35 min prior to the scan, participants were also asked to hold a lit cigarette (smoking-related cue) or sharpen a pencil in the presence of a lit candle (neutral cue condition). Fifty-five minutes prior to the scan (at baseline), and immediately after, each cue presentation (cue 1, cue 2, cue 3, cue 4), participants were asked to complete the Tobacco Craving Questionnaire (TCQ^33^) and Questionnaire on Smoking Urges-Brief (QSU-B^34^). The PANAS Positive and Negative Affect Schedule (PANAS^35^) was administered only at baseline, at the start of the PET session (cue 2) and 30 min relative to the start of the PET scan (cue 4). Vital signs (blood pressure and heart rate) were also taken at these times, and saliva was collected for the measurement of cortisol levels.

### PET image acquisition

The radiosynthesis of [^11^C]-(+)-PHNO has been described in detail elsewhere^36^. PET scans were performed using a Siemens-Biograph HiRez XVI (Siemens Molecular Imaging, Knoxville, TN, USA) PET/CT camera system, which measures radioactivity in 81 brain sections with a reconstructed pixel size of 1.07 × 1.07 × 2.00 mm each with an in-plane resolution of 5 mm full-width at half maximum (FWHM). A CT transmission scan was acquired for attenuation correction. The PET emission scan, acquired in 32-bit list mode, began with the slow bolus injection of [^11^C]-(+)-PHNO (duration of the bolus injection approximately 2 min). Emission data were reconstructed by 2D filtered back projection to yield dynamic images with 15 1-min frames and 15 5-min frames. The emission scan lasted for 90 min. The raw data were reconstructed by filtered-back projection. A custom-fitted thermoplastic mask (Tru-Scan Imaging, USA) was made for each subject to reduce movement during the acquisition. A total of 370 ± 40 MBq (approximately 10 ± 1 mCi) of [^11^C]-(+)-PHNO was injected as a bolus into an antecubital vein.

### MRI image acquisition

Subjects underwent standard proton density weighted brain magnetic resonance imaging (MRI) on a Discovery MR750 3 T MRI scanner (General Electric, 3 T MR750) (slice thickness 2 mm; interleaved; slice number, 84; repetition time, 6000 ms; echo time, 8 ms; number of excitations, 2; acquisition matrix, 256 × 192; FOV, 22 × 16.5 cm) to aid region of interest delineation of the PET images.

### PET image analysis

#### Region of interest (ROI)-based analysis

ROI delineation and time activity curve analyses were performed using the ROMI software (details in^37^). Functional sub-compartments of the striatum^38^ including the associative striatum (AST), limbic striatum (LST), and sensorimotor striatum (SMST) were chosen as ROIs. Delineation for the globus pallidus (GP; whole), ventral pallidum (VP) and substantia nigra (SN) is described elsewhere^39^.

#### Non displaceable binding potential

[^11^C]-(+)-PHNO non displaceable binding potential (BP_ND_) was estimated in each ROI using the simplified reference tissue method^40^ (SRTM), with cerebellar cortex (excluding vermis) as reference region. Parameter estimation was performed using PMOD (Version 2.8.5; PMOD Technologies Ltd, Zurich, Switzerland).

### Statistical analyses

[^11^C]-(+)-PHNO BP_ND_ was analysed with a Cue (neutral, smoking) X ROI (SN, VP, GP, LST, AST. SMST) ANOVA with Bonferroni-corrected two-tailed paired samples *t*-tests on the effect of Cue separately for each ROI. The TCQ was analysed as four subscales (TCQ1, TCQ2, TCQ3, TCQ4), and scores for the subscales of QSU1, QSU2, PANAS positive and PANAS negative were computed.

Scores for baseline (pre-cue) presentation for the TCQ1, TCQ2, TCQ3, TCQ4, QSU1, QSU2, PANASpos and PANASneg were subtracted from each of the four cue (neutral/smoking) presentations to obtain a difference score for each of the 4 cue presentations. These difference scores for the neutral cue were subtracted from these difference scores for the smoking cue (smoking-neutral) to obtain a difference score representing the difference between the smoking and neutral cues.

Questionnaire data were analysed with a repeated-measures ANOVA on the effect of Time (baseline, cue 1, cue 2, cue 3, cue 4) separately for each of the TCQ1, TCQ2, TCQ3, TCQ4, QSU1, QSU2. The PANASpos and PANASneg were also analysed with a repeated-measures ANOVA on the effect of Time (baseline, cue 2, cue 4).

Data was analysed with SPSS 25.0. An alpha of 0.05 was adopted for all analyses. The Geisser-Greenhouse correction was used where a significant Mauchley’s test of sphericity was revealed.

## Data Availability

The datasets generated during and/or analysed during the current study are available from the corresponding author on reasonable request.
